# High circulating osteoprotegerin levels are associated with non-zero blood groups

**DOI:** 10.1186/s12872-016-0287-2

**Published:** 2016-05-26

**Authors:** Elod Erno Nagy, Timea Varga-Fekete, Attila Puskas, Piroska Kelemen, Zoltan Brassai, Katalin Szekeres-Csiki, Timea Gombos, Maria Csilla Csanyi, Jolan Harsfalvi

**Affiliations:** Department of Pharmaceutical Biochemistry, University of Medicine and Pharmacy, Targu-Mures, Romania; Clinical County Hospital, Targu-Mures, Romania; IInd Clinic of Internal Medicine, University of Medicine and Pharmacy, Targu-Mures, Romania; Clinical Research Centre, University of Debrecen, Debrecen, Hungary; IIIrd Department of Internal Medicine, Semmelweis University Budapest, Budapest, Hungary; Department of Biophysics and Radiation Biology, Semmelweis University Budapest, Faculty of Medicine, H-1444 Budapest, P.O.B. 263, Hungary

**Keywords:** Osteoprotegerin, Von Willebrand factor, Peripheral arterial disease, Endothelial dysfunction, AB0 blood groups, Atherosclerosis, Atherosclerosis, Critical ischemia, Hypertension, Diabetes

## Abstract

**Background:**

Osteoprotegerin (OPG) and von Willebrand factor (VWF) form complex within endothelial cells and following secretion. The nature of blood group antigens strongly influences the levels of circulating VWF, but there is no available data concerning its ascendancy on OPG levels. We aimed to assess the relationship of AB0 blood groups with OPG, VWF levels (VWF: Ag) and collagen binding activity (VWF: CB) in peripheral arterial disease (PAD) patients.

**Methods:**

Functional and laboratory parameters of 105 PAD patients and 109 controls were examined. Results of OPG, VWF: Ag, VWF: CB (ELISA-s) were analysed by comparative statistics, together with clinical data.

**Results:**

OPG levels were higher in patients than in controls (4.64 ng/mL vs. 3.68 ng/mL, *p* < 0.001). Among patients elevation was marked in the presence of critical limb ischemia (5.19 ng/mL vs. 4.20 ng/mL, *p* = 0.011). The OPG in patients correlated positively with VWF: Ag and VWF: CB (*r* = 0.26, *p* = 0.008; *r* = 0.33, *p* = 0.001) and negatively with ankle-brachial pressure index (*r* = -0.22, *p* = 0.023). Furthermore, OPG was significantly elevated in non-0 blood groups compared to 0-groups both in patients and controls (4.95 ng/mL vs. 3.90 ng/mL, *p* = 0.012 and 4.09 ng/mL vs. 3.40 ng/mL, *p* = 0.002).

**Conclusions:**

OPG levels are associated to blood group phenotypes and higher in non-0 individuals. Increased OPG levels in PAD characterize disease severity. The significant correlation between OPG and VWF:CB might have functional importance in an atherothrombosis-prone biological environment.

**Electronic supplementary material:**

The online version of this article (doi:10.1186/s12872-016-0287-2) contains supplementary material, which is available to authorized users.

## Background

Peripheral arterial disease (PAD) is an occlusive manifestation of atherosclerosis involving most frequently the limbs. Persons with an increased risk are primarily the elderly, smokers, and patients with diabetes, hypertension and dyslipidaemia. The third important clinical manifestation of atherosclerosis presents significant clinical overlap with coronary artery disease (CAD) and carotid stenosis [1, 2]. Intermittent claudication is the leading symptom of PAD and a 10 year follow-up investigative branch of Framingham study showed that it has the lowest occurrence rate in individuals with blood group 0 [3].

Endothelial dysfunction is an early and important trigger of atherogenesis, emerging as a response to a number of various oxidative, infectious and mechanical injuries. Von Willebrand factor (VWF) and osteoprotegerin (OPG) are markers of these processes [4, 5].

VWF, a large multimeric glycoprotein that adheres to sub-endothelial collagen in order to catch bound platelets at high shear- and elongation forces [6]. Osteoprotegerin (OPG) is a dimeric glycoprotein with a multifunctional cytokine role and highly expressed in a large number of tissues [7]. They are associated in plasma, co-localized in, and co-secreted from the Weibel-Palade bodies of the endothelial cells [8, 9]. The up-regulation of OPG in severe forms of peripheral arterial disease has been described [10].

There is evidence that AB0 blood type is a major determinant of VWF [11-13] VWF level and activity were lower in 00, A0, B0, A^2^0, A^3^0 and rare genotypes than in AA, BB, and AB [14]. The relationship between circulating OPG levels and AB0 blood groups has never been analysed. Little is known about the potential action of glycosyltransferases A and B on OPG levels, the effects of glycosylation sites D178 and D183 on the formation of disulphide bonds, the correct folding of the molecule and how OPG glycosylation could interfere with degradative pathways [15–17].

Our hypothesis is, that AB0 blood group type is determinant of OPG levels in blood, similar to its role on VWF.

The aim of our observational study was to investigate the association of OPG and PAD along with VWF level (VWF: Ag) and collagen binding activity (VWF: CB) in an AB0 group dependent manner. To achieve our target, we performed measurement of serum and plasma OPG, VWF: Ag and VWF: CB along with AB0 blood group determination; then we analysed the relationship between those parameters in PAD patient and control groups; we then analysed their clinical and biochemical correlations. Since some studies have measured OPG from sera, while others focus on the plasma fraction [18, 19] and as the sample for VWF is plasma, we compared OPG results from serum and plasma too in order to test the relevance of OPG in this aspect.

## Methods

### Patients, diagnosis, risk factors and comorbidities

One hundred fifteen patients – previously diagnosed with various stages of PAD- were selected for this study. After excluding 10 patients with malignancies, acute inflammatory disorders, infections, or autoimmune vascular disease, 84 males and 21 females (age 64.5 ± 0.98 years) remained. The control group consisted of 109 individuals (75 males, 34 females, age 57 ± 1.34 years), selected from the National Health Care Survey, Romania. Before admission, written consignment was obtained from each patient; the study was approved by the ethics committee of the Clinical Emergency Hospital of Târgu-Mureș and was carried out in accordance with the Declaration of Helsinki for experiments involving humans.

Diagnosis of PAD was stated on medical history, clinical examination and measurement of the ankle-brachial pressure index (ABI) and Duplex ultrasonography. For calculation of the ABI, systolic blood pressure measurements were performed on the brachial, dorsalis pedis, and posterior tibial arteries bilaterally. The higher ankle pressure of the dorsalis pedis artery and the posterior tibial artery measurements for each limb were divided by the higher of the two brachial values. Both the lower and the mean of the two indexes were used for statistical comparison.

Intermittent claudication was assessed using the Walking Impairment Questionnaire. Stages III and IV, the presence of rest pain and/or ulceration associated with an ankle systolic pressure of ≤50 Hgmm were considered as critical leg ischemia.

Coronary artery disease was assumed to be present in patients with clinical history of angina pectoris or documented myocardial infarction (MI), previous positive coronary angiography or angioplasty, and/or resting ECG changes suggestive for ischemia: ST-segment depression, Q-wave changes or T-wave changes, according to the Minnesota coding method.

A Colour Duplex carotid artery scan was performed using a GE Agilent Image Point HXB.1 Sonos 4500/5500B.1 ultrasound system. B-mode, colour- and pulsed-wave Doppler analyses were performed on both sides to identify arterial wall lesions and stenosis (expressed as the percentage decrease in artery diameter). Where no plaques were identified, carotid intima-media thickness (CIMT) was measured bilaterally at the far wall of the distal common carotid artery, immediately proximal to bifurcation, in the end diastole. Carotid artery atherosclerosis was defined as CIMT > 1.00 mm, or the presence of plaques at any levels.

The risk factors evaluated in this study included diabetes mellitus, hypertension, haemostatic, lipid and inflammatory factors. Hypertension was diagnosed according to JNC 7 protocols. Diabetes mellitus was defined as fasting glucose >6.94 mmol/L on at least two measurements, or evidence for current use of insulin or oral hypoglycaemic medication.

### Laboratory analysis

Blood samples were taken after overnight fasting into vacutainers with either no additive or 3.2 % trisodium citrate (Becton-Dickinson Vacutainer Systems, UK). Centrifugation was performed at 3000 rpm for 10 min, after which the serum and plasma aliquots were separated, submitted to routine biochemistry or deep-frozen at −70 °C. Serum fasting glucose, total cholesterol, high-density cholesterol and triglycerides were determined by enzymatic methods on an ABBOTT AEROSET automated biochemistry analyser (Abbott Diagnostics, USA). High-sensitivity CRP (hs CRP) was measured on a COBAS INTEGRA 400 analyser by an immunoturbidimetric method (Roche Diagnostics, Switzerland). Plasma fibrinogen was determined according to the Clauss method with Fibrinogen Reagent (Technoclone, Austria) and Behnk Thrombotimer (Germany). AB0 blood group antigens were determined by the direct Beth-Vincent method with anti-A, anti-B, anti-AB sera (DiaClon AB0, Bio-Rad Laboratories, Switzerland).

VWF: Ag was measured by ELISA according to a previously established protocol [20], VWF: CB was measured as Ag, but type III collagen (Sigma, USA) was used for coating high binding capacity plate (Greiner Bio-One, Austria). Infiniti 200 M (Tecan Trading AG, Männedorf, Switzerland) was used for optical reading and its Magellan software for data processing. All samples were determined in duplicate and calculations were based on four-parameter Marquardt standard curve fitting. A standard was purchased from Siemens, Germany, and results were expressed as %.

OPG levels were determined both from serum and plasma using the R&D Systems DuoSet ELISA kit (DY805, R&D Systems Europe, UK), according to the manufacturer’s instructions. Recombinant human OPG (Part No. 840371; R&D Systems) served as standard, and concentrations were expressed as ng/mL. There are controversial results with regards to the relation of OPG serum and plasma levels in the literature. Different anticoagulants, clot activator, anti-proteases, time between-collection, centrifugation and storage cause the main variations [18, 19]. Bland-Altman analysis did not reveal difference between our serum and plasma based OPG measurements (*n* = 181, bias = 0.055, SD = 0.39), for this reason we used the plasma values for further statistical analysis. Intra-observer coefficient of variation for OPG, VWF: Ag and VWF: CB, the outcome parameters were below 11, 10 and 12 %, respectively.

### Statistical analysis

We used the IBM SPSS Statistics for Windows, Version 19.0 (IBM Corp., Armonk, NY, USA) and GraphPad Prism 6 (GraphPad Software Inc., USA) for the statistical calculations. Since the main parameters, OPG and VWF had a non-Gaussian distribution, we applied non-parametric statistical tests in our evaluation. The Kruskal-Wallis ANOVA was used for multiple group comparison; paired contrasts were carried out using the Mann-Whitney U test. Fisher’s exact test was applied to compare 2×2 or larger between-group distributions of discrete parameters. All correlations were performed by the Spearman rank-correlation test. Multiple linear regression models were applied to define the effect of clinical and laboratory variables on ln-transformed OPG levels.

## Results

### The main characteristics of the patients and control groups

First we show the clinical features followed by the biochemical characteristics of the patient and the control groups in Tables 1, labelling the results of the statistical comparisons.

**Table 1:** The main clinical and laboratory parameters of the patient and control groups

Parameters	Patients (*n* = 105)	Controls (*n* = 109)
Age ***	64.2 ± 0.98	57.0 ± 1.34
Gender (male)	84 (80)	75 (69)
Diabetes	32 (30.5)	23 (21.1)
Hypertension ***	92 (87.6)	44 (40.4)
Stroke history	12 (11.4)	6 (5.5)
Myocardial infarction history	11 (10.4)	18 (16.5)
CAD***	72 (68.6)	40 (36.7)
Intermittent claudication	105 (100)	no
Critical limb ischemia	30 (28.6)	no
Significant carotid stenosis ^a^	10 (13.5)	nd
Multiple atherosclerotic involvement***	21 (20)	4 (3.7)
Haemorheological treatment	105 (100)	no
Antihypertensives ***	87 (82.8)	29 (26.6)
Anticoagulants **	25 (23.8)	10 (9.2)
Statins ***	73 (69.5)	10 (9.2)
Fibrates	10 (9.5)	4 (3.7)
Rate of AB0 blood groups (0/non-0)	0.38	0.49
ABI (lower)	0.48 (0.36–0.60)	nd
ABI (mean)	0.59 (0.43–0.73)	nd
CIMT (lower) ^a^	1.00 (0.70–1.30)	nd
CIMT (mean) ^a^	1.25 (0.7–1.45)	nd
OPG [ng/mL] ***	4.64 (3.49–6.54)	3.68 (2.92–4.81)
VWF:Ag [%]	129 (96–174)	116 (94–150)
VWF:CB [%] *	123 (94–163)	107 (88–136)
Fibrinogen [g/L] ***	4.10 (3.04–5.03)	3.00 (2.32–3.50)
Total cholesterol [mmol/L]	4.83 (3.98–5.87)	5.10 (4.29–5.88)
HDL–cholesterol [mmol/L] ***	1.19 (1.03–1.40)	1.52 (1.28–1.74)
Triglycerides [mmol/L]	1.33 (0.96–1.76)	1.20 (0.86–1.74)
CRP [mg/L]	5.03 (2.22–9.67)	4.44 (1.84–6.55)

Age is expressed as mean ± SE, gender as the number of males and their percentage, disease states as number and percentage in brackets, compared to all individuals of the group, parameters are given as median with quartile range in brackets. * *p* < 0.05, ** *p* < 0.01, *** *p* < 0.001 significances of patients to control (Mann–Whitney U test for continuous variables and Fisher’s exact test for categorical variables), ^a^examined in 74 cases, no = no sign or history at controls, nd = not determined

The distribution of genders, incidence of diabetes, history of stroke and MI and the rate of 0/non-0 blood groups revealed no different between patients and controls. The age was higher; hypertension, CAD, multiple atherosclerotic involvement, antihypertensive, anticoagulant and statin usage were more frequent in the PAD group. ABI and CIMT were measured for diagnostic and staging purposes during hospitalization of PAD patients.

Results of the biochemical tests show that OPG levels [ng/mL] and VWF: CB [%] were significantly higher in patients than in controls (4.64 vs. 3.68, *p* < 0.001, and 123 vs. 107, *p* = 0.03). On the whole population, the relative risk of having PAD in the upper OPG quartile vs. the lower quartile was 1.75 (95 % CI: 1.17–2.60, *p* = 0.006). Fibrinogen levels were also significantly increased, while HDL-cholesterol levels were decreased in patients compared to controls, see Table 1.

### Correlations of OPG and the main clinical and laboratory parameters

OPG proved to be dependent on age (*r* = 0.36, *p* < 0.001) but independent of gender: 4.02 (3.09–5.55) in males vs. 4.45 (3.48–6.55) in females (*p* = 0.093). Diabetic patients showed higher OPG values than non-diabetics with a borderline significance, 4.83 (4.09–7.80) vs. 4.48 (3.30–6.26), *p* = 0.052. OPG levels were significantly elevated in the whole population (patients and controls) with hypertension (*p* < 0.001), previous stroke (*p* = 0.005), CAD (*p* = 0.037), anti-hypertensive (*p* < 0.001) and fibrate (*p* = 0.033) users. OPG levels were elevated but not significantly in MI history positive individuals, anticoagulant and statin receivers. Among patients, those with critical limb ischemia (*n* = 30) had a significantly higher OPG and VWF: CB, than the rest of the group (5.19 vs. 4.20, *p* = 0.011, and 138 vs. 116, *p* = 0.031). In patients OPG showed significant negative correlations with the mean and lower ABI values (*r*= -0.22, *p* = 0.023 and *r* = -0.21, *p* = 0.032). OPG was unrelated to CIMT obtained on patients with no significant carotid stenosis (data not shown).

**Fig. 1:**
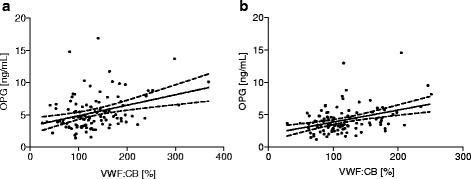
Correlation between plasma OPG levels and VWF: CB activities. **a** Correlation of OPG with VWF: CB in patients. **b** Correlation of OPG with VWF: CB in controls. Mean ± 95 % confidence intervals are shown by the regression- and the dotted lines

OPG correlated positively with VWF: Ag and VWF: CB (*r* = 0.26, *p* = 0.008; *r* = 0.33, *p* = 0.001, respectively) in patients and also in controls (OPG-VWF: Ag *r* = 0.25, *p* = 0.008, OPG-VWF: CB *r* = 0.26, *p* = 0.005). Figure 1a and b show the correlation and linear regression line with 95 % confidence intervals for correlation of OPG and VWF: CB in patients and in controls. There was no significant relationship between OPG, fibrinogen, total and HDL-cholesterol, triglycerides and CRP levels.

We did not test the effect of smoking on OPG, as at the time of admission no patients were active smokers.

### Clinical and laboratory parameters of zero- and non-zero blood group patients and controls

Comparison of clinical and laboratory parameters for 0- and non-0 blood group patients and controls is given in Additional file 1: Table S1. Age, distribution of genders, incidence of diabetes, hypertension, stroke history, CAD, critical limb ischemia, carotid stenosis were not significantly different between groups. History of previous MI was assessed significantly more often in the non-0 group. The positive history for MI was significantly related to the presence of the non-0 blood groups: all 11 patients and 16 controls from 18 with a previous MI had an A, B or AB phenotype (difference in proportions *p* = 0.002 for the overall population and = 0.031 for both patients and controls). Intima-media thickness showed a mild, but non-significant increase in the 0 compared to non-0 group, the frequency of carotid stenosis in the two groups was similar. CIMT and ABI values, fibrinogen, total serum cholesterol, HDL-cholesterol, triglycerides, CRP levels showed no difference.

**Fig. 2:**
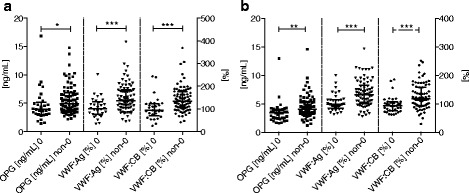
Plasma OPG, VWF: Ag levels and VWF:CB activity in patients and controls. Column scatter plot graph of the OPG and VWF values in patients (**a**) and controls (**b**) according to 0 and non-0 blood groups. Values represented as median with interquartile range. Significant differences marked with * for *p* < 0.05, ** *p* < 0.01, *** *p* < 0.001

Comparison of Non-0 and 0 patients presented significantly higher OPG (4.95 vs. 3.90, *p* = 0.012), VWF: Ag (137 vs. 102, *p* < 0.001) and VWF: CB (134 vs. 94, *p* < 0.001), as shown in Fig. 2. These findings were also characteristic of controls, as follows: OPG (4.09 vs. 3.40, *p* = 0.002), VWF: Ag (132 vs. 98, *p* < 0.001), VWF: CB (117 vs. 93, *p* < 0.001).

Moreover, we observed that the nature of blood groups markedly influenced the OPG-VWF: CB correlation. In the 65 patients and controls with group 0, the correlation was absent (*r* = 0.017, *p* = 0.891). In non-0 patients and controls (*n* = 149), OPG correlated significantly with VWF: CB (*r* = 0.28, *p* < 0.001).

### Association between circulating OPG and ABzero blood groups

A number of 29 blood group 0 patients had been registered (27.6 %), while the non-0 group had the following composition: 46 group A (43.8 %), 18 group B (17.1 %), 12 group AB (11.4 %) see Additional file 2: Table S2. Among controls, the distribution of AB0 phenotypes was as follows: 36 (33 %) group 0, 32 (29.3 %) group A, 29 (26.6 %) group B, 12 (11 %) group AB. The OPG VWF levels and collagen binding activity by individual blood groups are detailed in Additional file 2: Table S2. OPG showed the highest levels at blood group AB in patients and at group A in controls, while VWF: Ag and CB were highest in group B and AB, respectively.

Due to the relatively small number of patients and controls included in each blood group, we weighed the effect of AB0 groups as 0- and non-0.

### Multiple linear regression model for high OPG levels

On the basis of the aforementioned results we set up a multiple linear regression model for the determinants of high OPG values Table 2. The regression model describes the ln-transformed OPG levels as a dependent variable in the whole studied population, and contains gender, age, diagnosis of PAD, stroke history, ln-transformed VWF: CB and 0/non-0 blood group categories. VWF: CB, PAD, AB0 categories remained significantly associated with the OPG levels after adjustment for diabetes, hypertension, MI, CAD, anti-hypertensive, anti-coagulant drugs, statin and fibrate usage.

**Table 2:** Multiple linear regression model for ln-transformed OPG levels in the overall study population

	B	SE of B	*p*-value
Gender	0.219	0.065	<0.001
Age	0.007	0.002	0.002
Presence of PAD	0.168	0.057	0.004
Stroke history	0.216	0.101	0.034
VWF:CB	0.238	0.075	0.002
0/non-0 groups	0.156	0.064	0.016

*R* = 0.519, the adjusted R square of the model is 0.248, *p* < 0.001

Despite the strong association with the non-0 blood groups, a positive history for MI did not prove to be a significant determinant of OPG levels.

## Discussion

The biological consequence of elevated OPG remains a subject of debate, while it is has been shown for several years that the circulating OPG concentration correlates with the severity of atherosclerotic conditions [7]. Although the higher prevalence of PAD among non-0 blood group carriers has been recognized for a long time [21], association of OPG and AB0 blood groups has not been analysed until our present study.

In our design, known peripheral arterial disease patients were selected with a high frequency of multiple atherosclerotic involvement and comorbidities, such as hypertension and diabetes. The clinical and laboratory characteristics of this group were compared to a control group. Diabetes, hypertension, stroke history, myocardial infarction history and CAD occurred also in the control group that in contrast, did not express PAD.

Considering the mean ABI values, our patient group had advanced forms of atherosclerosis. OPG values showed significant negative correlation with the mean and lower ABI, which are measures of PAD severity. Corroborating previous results, we observed significantly higher values of OPG in patients compared to individuals without PAD [22–24]. The association of OPG with PAD is still controversial, since some authors have failed to prove it [25, 26].

We also elucidated a higher OPG in sufferers of critical limb ischemia and a negative correlation with the ABI scores. In the study of Ziegler et al., plasma OPG correlated with the ABI, however, the authors found no significant increase in patients compared to controls. It should be noted that they did not determine the AB0 blood groups, which, in the cases of their 67 patients and 94 controls, could have influenced the results. The incidence of diabetes, hypertension, the level of plasma fibrinogen, total and HDL-cholesterol were comparable to the characteristics of our group [25]. Ali et al. documented the association between serum OPG and ABI [22]. In this study, two separate cohorts were included: one consisting of Afro-Americans and another of non-Hispanian whites, predominantly women, with a 73–80 % incidence of hypertension, high BMI values, but low frequency of peripheral arterial disease (9 % overall).

There are some controversial results regarding the effect of diabetes and hyperglycaemic status on OPG levels: absence of relationship with the glycaemia status [27], significantly elevated OPG in diabetic nephropathy and in Japanese men with diabetes type 2 [28], slightly increased OPG values in gestational diabetes [29]. In our observations, the accompanying diabetes had only a borderline effect on OPG levels.

OPG has been defined as an important mediator of vascular calcification and is influenced by the presence of arterial hypertension [7, 30]. In our whole population (but not in PAD patients) high OPG levels were associated with the presence of hypertension.

Our findings revealed an association between higher OPG levels and CAD in the whole studied population, which was not significant in the patients with the leading symptoms of PAD. A number of clinical studies mention that OPG correlates to the presence and the most important cardiovascular disease endpoints, such as long-term mortality in CAD patients [31]. In our MI history patients, OPG was higher but the difference did not reach the threshold of statistical significance due to the reduced number of individuals in the subgroup. Acute coronary events were correlated with elevation of circulating OPG, however, some studied failed to link myocardial ischemia to high OPG [32]. CAD also causes increased levels of OPG, however, in polyvascular disease with no acute myocardial events, this association might be masked by the advanced peripheral involvement and calcification. Patients with carotid stenosis show variable OPG levels, with elevations especially on diabetes type 2 backgrounds [33]. In our study, the subgroup with considerable carotid stenosis showed no higher OPG levels than the rest of the patients. This was also found in cases with a previous stroke history. In both subgroups OPG proved to be higher than in those without significant stenosis or stroke, but due to their restricted size the difference did not reach significance.

We observed positive correlations between OPG, VWF: Ag and VWF: CB both in patients and controls. This significant relationship has not been described before. In our setting the VWF: CB shows slightly stronger association with OPG levels, than VWF: Ag. In endothelial cells, OPG resides in the Weibel-Palade bodies, together with VWF and P-selectin and is co-secreted in complex with VWF [8]. Vinholt et al. recently developed an ELISA to measure it in plasma and found that only a small fraction of OPG circulates in complex, and this amount cannot be related to the severity of coronary calcification. This finding is important but does not rule out the functional importance of OPG-VWF complexes [34]. According to our results, the correlation of OPG and VWF: CB activity is significant only in non-0 group individuals. The number of individuals in 0 blood groups is relatively small to result in a significant correlations. It is not known if OPG has ABH antigenic structure. VWF does have, but it is also not known if the A and B antigenic forms of it bind better OPG than H form. The presence of non-0 blood groups might confer to carriers an increased potential for atherothrombosis [13]. Recently, further relationships of VWF and OPG were given by Rutten et al., who measured the “active VWF” fraction and highlighted that in 1026 first ST-segment elevation myocardial infarction (STEMI) cases the plasma medians of total VWF, “active VWF”, OPG and the ratio of VWF to “a disintegrin and metalloproteinase with a thrombospondin type 1 motif, member 13” (ADAMTS-13) were at a significantly high risk for STEMI. After adjustments of these, only the “active VWF” remained significant. In this compilation out of the Chinese, Scottish and Italian cohort: only the latter displayed elevated OPG, and AB0 blood groups were not determined [35].

The most important finding of our study is that we evidenced a significant association of high OPG with the non-0 blood groups in PAD patients and controls. The A and AB blood group carriers presented the highest OPG values both in patients and controls (Additional file 1: Table S1). VWF: Ag and VWF: CB levels showed similar differences in favour of non-0 groups. The correlation between VWF: CB and the categories of AB0 group is noteworthy. Specifically VWF: CB is sensitive for the size of the VWF multimers [36], but we have not found any information concerning the relation of VWF multimer distribution and AB0 group.

The effects of blood group antigens on atherosclerosis manifestations, disease progression and outcomes have been extensively studied [12, 37]. The glycosylated residues present on VWF at position N1574 influence the cleavage of the multimers by ADAMTS-13 [38]. Whether VWF glycosylation affects or not complex formation between OPG and VWF is a question for further study.

To our knowledge, we are the first to show the association of circulating OPG levels with AB0 antigens. In addition, our results show a statistically strong difference between the VWF: CB activity of 0 and non-0 group patients.

Ziegler et al. suggest a decreased OPG clearance in PAD, which seems feasible for the Fontaine III-IV stages [25]. In the mirror of our results, we assume that non-0 blood group carbohydrate antigens present on the molecule might influence OPG levels, either by inhibition of protease cleavage or by an enhanced co-secretion along VWF from a triggered vessel-wall with endothelial dysfunction.

One of the limitation of our study was that the data presented here are descriptive in nature and the observed associations do not prove causal relationships. The other limitation was the relatively restricted number of patients enrolled, and the lack of AB0 genotyping possibilities, which could offer a more exact assessment of the correlations described. Although we found evidence for the association of OPG with 0 and non-0 blood group, further studies are needed to confirm this relationship in larger populations.

## Conclusion

High OPG levels are characteristic of PAD, especially in patients with critical limb ischemia. Our study brings new data regarding the association of high OPG values with non-0 AB0 blood groups in PAD patients. While the relationship of high VWF with non-0 groups is well characterized and thought to have a causal role in the higher incidence of atherothrombotic events in comparison to 0 groups, we are the first to correlate high circulating OPG with these determinants. It is also new that VWF collagen binding activity is a strong predictor of OPG levels and that the OPG-VWF correlation is AB0 phenotype dependent. We think VWF and OPG are markers of endothelial perturbation or damage as a result of arterial disease. To elucidate whether elevated OPG represents an increased prothrombotic potential or, on the other hand, a protective, tackling actor in peripheral arterial disease with high atherosclerotic burden, needs further studies.

## Abbreviations

ABI, ankle-brachial index; ADAMTS-13, a disintegrin and metalloprotease with thrombospondin-1 repeats; AP, angina pectoris; CAD, coronary artery disease; CIMT, carotid intima-media thickness; hsCRP, high-sensitive C-reactive protein; MI, myocardial infarction; OPG, osteoprotegerin; PAD, peripheral arterial disease; STEMI, first ST-segment elevation myocardial infarction; VWF, Von Willebrand Factor; VWF: Ag, VWF antigen; VWF: CB, VWF collagen binding.

## Additional files

Additional file 1: Table S1. Clinical and laboratory parameters of patients and controls by 0/non-0 blood groups. (DOCX 24 kb) Additional file 2: Table S2. OPG and VWF in patients and controls by AB0 groups. (DOCX 21 kb)
