# Supplementary material for: High circulating osteoprotegerin levels are associated with non-zero blood groups
Source: BMC Cardiovasc Disord. 2016 May 26;16:106. doi: 10.1186/s12872-016-0287-2 (PMC4937555; doi:10.1186/s12872-016-0287-2)
Supplement: Additional file 1: Table S1. — Clinical and laboratory parameters of patients and controls by 0/non-0 blood groups. (DOCX 24 kb) [file 12872_2016_287_MOESM1_ESM.docx]

**Table S1**. Clinical and laboratory parameters of patients and controls by 0/ non-0 blood groups

| **Parameters** | **Blood Group** | | |  |
| --- | --- | --- | --- | --- |
|  | **Patients (n=105)** |  | **Controls (n=109)** |  |
|  | **Group 0** | **Groups non-0** | **Group 0** | **Groups non-0** |
|  | **(n=29)** | **(n=76)** | **(n=36)** | **(n=73)** |
| Age | 64.76 ± 1.65 | 64.03 ± 1.19 | 53.36 ± 2.54 | 58.69 ± 1.56 |
| Gender (male) | 25 (86.2) | 59 (77.6) | 23(63.9) | 52 (71.2) |
| Diabetes | 9 (31) | 23 (30.2) | 6 (16.6) | 17 (23.8) |
| Hypertension | 26 (90) | 66 (87) | 9 (25)* | 35 (47.9)* |
| Stroke history | 3 (10.3) | 9 (11.8) | 0 (0) | 6 (8.2) |
| Myocardial infarction history | 0 (0)* | 11 (14.5)* | 2 (5.5)* | 16 (21.9)* |
| CAD | 19 (65.5) | 53 (69.7) | 9 (25) | 31 (42.5) |
| Critical limb ischemia | 9 (31) | 21 (27.6) | no | no |
| Significant carotid stenosis † | 3 (13.6) | 7 (13.4) | nd | nd |
| Multiple atherosclerotic involvement | 3 (10.3) | 18 (23.7) | 0 (0) | 4 (5.5) |
| Hemorheological treatment | 29 (100) | 76 (100) | no | no |
| Antihypertensives | 24 (82.7) | 63 (82.9) | 6 (16.7) | 22 (30.1) |
| Anticoagulants | 8 (27.6) | 17 (22.4) | 0 (0) | 6 (8.2) |
| Statins | 20 (68.9) | 53 (69.7) | 3 (8.3) | 7 (9.6) |
| Fibrates | 4 (13.8) | 6 (7.9) | 2 (5.5) | 2 (2.7) |
| CIMT (mean) [mm] † | 1.22 (0.71-1.55) | 1.25 (0.7-1.45) | nd | nd |
| CIMT (lower) [mm] † | 1.05 (0.71-1.37) | 0.98 (0.7-1.3) | nd | nd |
| ABI (mean) | 0.68 (0.39-0.8) | 0.57 (0.46-0.73) | nd | nd |
| ABI (lower) | 0.48 (0.35-0.68) | 0.48 (0.35-0.58) | nd | nd |
| Fibrinogen [g/L] | 4.35 (3.12-5.36) | 4.09 (3.04-4.84) | 2.70 (2.23-3.09)* | 3.11 (2.40-3.65)* |
| Total cholesterol [mmol/L] | 4.96 (4.29-5.79) | 4.81 (3.98-5.92) | 5.17 (4.31-5.94) | 4.99 (4.11-5.81) |
| HDL-cholesterol [mmol/L] | 1.24 (0.96-1.50) | 1.19 (1.06-1.37) | 1.47 (1.26-1.70) | 1.57 (1.29-1.73) |
| Triglycerides [mmol/L] | 1.33 (0.96-1.75) | 1.33 (0.97-1.78) | 1.04 (0.62-1.73) | 1.30 (0.95—1.87) |
| CRP [mg/L] | 5.23 (2.74-13.08) | 5.03 (1.72-8.88) | 2.67 (0.97-5.54)* | 4.75 (2.31-8.77)* |

Age is expressed as mean ± SE, gender as the number of males and their percentage, disease states expressed as number and percentage in brackets, compared to all individuals in the group, laboratory parameters are given as median and quartile range in brackets. † examined in 25 (0) and 59 (non-0) cases, respectively. no= no sign or history at controls, nd= not determined .* P values presented for Mann-Whitney U test for continuous variables and Fisher’s exact test for categorical variables. Significant differences of 0 vs. non-0 group comparisons marked with * for p<0.05.
