# Supplementary material for: High circulating osteoprotegerin levels are associated with non-zero blood groups
Source: BMC Cardiovasc Disord. 2016 May 26;16:106. doi: 10.1186/s12872-016-0287-2 (PMC4937555; doi:10.1186/s12872-016-0287-2)
Supplement: Additional file 2: Table S2. — OPG and VWF in patients and controls by AB0 groups. (DOCX 21 kb) [file 12872_2016_287_MOESM2_ESM.docx]

**Table S2**. OPG and VWF in patients and controls by AB0 groups

**A**. Patients

| **Parameters** | **Group 0**  **n=29** | **Group A**  **n=46** | **Group B**  **n=18** | **Group AB**  **n=12** |
| --- | --- | --- | --- | --- |
| OPG [ng/mL] | 3.90 (3.02-4.94) | 4.95 (3.63-6.55)* | 4.92 (3.71-7.77) | 5.16 (3.99-7.05) |
| VWF:Ag [%] | 102 (77-129) | 136 (106-178)** | 155 (115-195)*** | 129 (90-173) |
| VWF:CB [%] | 94 (76-122) | 128 (99-164)** | 159 (124-188)*** | 127 (97-154) |

**B**. Controls

| **Parameters** | **Group 0**  **n=36** | **Group A**  **n=32** | **Group B**  **n=29** | **Group AB**  **n=12** |
| --- | --- | --- | --- | --- |
| OPG [ng/mL] | 3.40 (2.45-4.00) | 4.47 (3.34-5.39)** | 3.69 (3.26-5.32)* | 3.95 (3.21-5.34)* |
| VWF:Ag [%] | 98 (86-113) | 123 (91-150) | 129 (112-162)*** | 152 (120-206)*** |
| VWF: CB [%] | 93 (76-107) | 108 (90-140)* | 117 (101-143)*** | 150 (106-177)*** |

Values expressed as median, with lower-to-upper quartiles in brackets. * p<0.05, ** p<0.01, *** p<0.001 in comparison to group 0 values.
